# Vertical transmission of zika virus in *Aedes albopictus*

**DOI:** 10.1371/journal.pntd.0008776

**Published:** 2020-10-15

**Authors:** Zetian Lai, Tengfei Zhou, Jiayong Zhou, Shuang Liu, Ye Xu, Jinbao Gu, Guiyun Yan, Xiao-Guang Chen

**Affiliations:** 1 Department of Pathogen Biology, Key laboratory of Tropical Disease Research of Guangdong Province, School of Public Health, Southern Medical University, Guangzhou, China; 2 Program in Public Health, University of California, Irvine, Irvine, CA, United States of America; University of Glasgow, UNITED KINGDOM

## Abstract

**Background:**

Zika virus (ZIKV) is an arthropod-borne flavivirus transmitted by *Aedes* mosquitoes. *Aedes albopictus* is an important vector of ZIKV worldwide. To date, most experiments have focused on the vertical transmission of ZIKV in *Ae*. *aegypti*, while studies on *Ae*. *albopictus* are very limited. To explore vertical transmission in *Ae*. *albopictus*, a series of laboratory studies were carried out.

**Methodology/Principal findings:**

In this study, *Ae*. *albopictus* were blood-fed with ZIKV-infectious blood, and the ovaries and offspring viral infection rates were analyzed by reverse transcription PCR (RT-PCR), real-time reverse transcription PCR (RT-qPCR) and immunohistochemistry (IHC). ZIKV was detected in the ovaries and oviposited eggs in two gonotrophic cycles. The minimum filial egg infection rates in two gonotrophic cycles were 2.06% and 0.69%, and the effective population transmission rate was 1.87%. The hatching, pupation, and emergence rates of infected offspring were not significantly different from those of uninfected offspring, indicating that ZIKV did not prevent the offspring from completing the growth and development process. ZIKV was detected in three of thirteen C57BL/6 suckling mice bitten by ZIKV-positive F_1_ females, and the viremia persisted for at least seven days.

**Conclusions/Significance:**

ZIKV can be vertically transmitted in *Ae*. *albopictus* via transovarial transmission. The vertical transmission rates in F_1_ eggs and adults were 2.06% and 1.87%, respectively. Even though the vertical transmission rates were low, the female mosquitoes infected via the congenital route horizontally transmitted ZIKV to suckling mice through bloodsucking. This is the first experimental evidence of offspring with vertically transmitted ZIKV initiating new horizontal transmission. The present study deepens the understanding of the vertical transmission of flaviviruses in *Aedes* mosquitoes and sheds light on the prevention and control of mosquito-borne diseases.

## Author summary

Zika virus (ZIKV) is a mosquito-borne flavivirus that poses a serious threat worldwide because of its associated serious neurological complications, such as Guillain-Barré syndrome in adults and microcephaly in newborns. Vertical transmission of ZIKV in humans has been confirmed. Furthermore, there have been reports of ZIKV infection in field-collected eggs, larvae and adult mosquitoes, which implies that ZIKV can also be vertically transmitted in mosquito vectors. However, the characteristics of vertical transmission of ZIKV in *Aedes albopictus* remain unclear. Here, we infected mosquitoes by allowing them to feed on an infectious blood meal. F_1_ progeny (eggs and adults) from mosquitoes with ZIKV-positive ovaries were studied. Our results demonstrate that ZIKV can be vertically transmitted in *Ae*. *albopictus* via transovarial transmission. The female mosquitoes infected via the congenital route can horizontally transmit ZIKV to suckling mice through bloodsucking. These updated findings can be used for ZIKV disease prevention and vector control strategies.

## Introduction

Zika virus (ZIKV), a mosquito-borne flavivirus, is known globally because of its associated serious neurological complications, such as Guillain-Barré syndrome in adults [1] and microcephaly in newborns [2], which pose serious threats; thus, ZIKV has become a public health emergency of international concern [3]. It has been reported that after ZIKV infection during pregnancy, the virus replicates and crosses the placental barrier [4]. Vertical transmission is the main route of ZIKV-induced microcephaly in the fetus.

Many flaviviruses, including dengue [5], yellow fever [6], West Nile [7], and St. Louis encephalitis virus [8], can be transmitted vertically from infected female mosquitoes to their offspring, according to previous studies. There have been reports of the vertical transmission of ZIKV in mosquito vectors. Evidence of ZIKV infection in field-collected eggs and mosquitoes has been reported in different [9–13] countries. ZIKV-positive offspring have been reported in the literature, with different filial infection rates [14–17], while negative results in F_1_ offspring have also been reported [18–21]. In *Aedes aegypti*, the filial infection rate ranged from 0 to 6.99% [14–18]; in *Culex quinquefasciatus*, the filial infection rate was between 0.04% and 1.52% [16]; and the filial infection rate in *Ae*. *albopictus* ranged from 0 to 1.18% [14, 16, 19, 20]. These rates varied, and these contradictory reports may be due to different experimental designs, such as the intrathoracic inoculation of ZIKV or the testing of pooled larvae rather than the assessment of individual mosquitoes after ingestion of an infectious blood meal. At present, the characteristics of vertical transmission of ZIKV in mosquito vectors are not yet completely known. In addition, microcephaly is caused by the vertical transmission of ZIKV from an infected mother to her infant. Does the vertical transmission of ZIKV cause similar pathological effects in mosquito hosts?

*Ae*. *albopictus* is an important vector of ZIKV globally [14, 16, 19, 20] and a potential primary vector in China. To date, most experiments have focused on the vertical transmission of ZIKV in *Ae*. *aegypti*, while studies on other susceptible mosquitoes, *Ae*. *albopictus*, are very limited. To explore the questions above, a series of laboratory studies were carried out to characterize the vertical transmission of ZIKV in *Ae*. *albopictus*.

## Materials and method

### Ethics statement

C57BL/6 suckling mice (one day old) were purchased from the Animal Experimental Center of Southern Medical University, Guangdong Province, China. This study was carried out in accordance with the recommendations in the Guide for the Care and Use of Laboratory Animals of the National Institutes of Health. All animal experiments were performed under specific pathogen-free conditions. All mice received humane care and were given standard diet and water ad libitum. The protocol was approved by the Office of Laboratory Animal Welfare (approval number: A5867-01).

### Mosquitoes

An established laboratory colony of *Ae*. *albopictus* collected from Foshan, Guangdong Province, China, in 1981 was provided by the Guangdong Provincial Center for Disease Control and Prevention. The larvae (120–200 larvae/L water) were reared in stainless steel trays containing dechlorinated water and were fed yeast and turtle food. The mosquitoes were reared in an insectary, which was maintained at 27 ± 1°C with 70%–80% relative humidity and a 16-h light/8-h dark photoperiod, and offered a 10% sucrose solution ad libitum.

### Virus

The ZIKV strain (GenBank accession no. KU820899.2) used in the study was isolated from a patient in Zhejiang Province, China, in February 2016 and was classified as the Asian lineage [22, 23]. This strain was obtained after the second passage in *Ae*. *albopictus* C6/36 cells maintained at 28°C in Roswell Park Memorial Institute (RPMI) 1640 medium supplemented with 2% heat-inactivated fetal bovine serum. Cells grown in a 25-cm^2^ culture flask were inoculated with ZIKV at a multiplicity of infection (MOI) of 1. The culture flask was inoculated at 28°C for 4–5 days. The fresh virus suspension (5.45 ± 0.34 log_10_ copies/μl) was used to prepare the blood meal, which contained 0.8 ml of ZIKV and 0.4 ml of defibrinated sheep blood (Solarbio, Beijing, China), with a concentration of 4.2 ± 1.2× 10^5^ PFU/ml.

### Animals

C57BL/6 suckling mice (one day old) were purchased from the Animal Experimental Centre of Southern Medical University, Guangdong Province, China. Mice were breastfed by their own mothers. Weights of each nest of mice were measured at birth, and the differences between nests were not statistically significant (*p* > 0.05). The study design was approved by the Office of Laboratory Animal Welfare (approval number: A5867-01), and animal care was in accordance with institutional guidelines.

### Infection of mosquitoes and ZIKV detection

After emergence, the *Ae*. *albopictus* adults were kept in cages (20 cm × 20 cm × 35 cm) for mating in the next 5 days. Female mosquitoes were starved for 24–48 h and were infected by allowing them to feed on an infectious blood meal through a Hemotek blood feeding system (Discovery Workshops, Lancashire, UK). Fully engorged females were incubated under insectary conditions as mentioned above. The experiments were conducted in a biosafety level 2 laboratory.

The midguts and ovaries of 10 mosquitoes were dissected at each of the time points during the first gonotrophic cycle (FGC), corresponding to 4, 7, 10, and 14 dpi. The tissues were cold homogenized separately after immersion in 50 μl of TRIzol [24]. Total RNA extraction was performed according to the manufacturer’s protocol of TRIzol reagent. The total RNA products were dissolved in 20 μL of RNase-free water. ZIKV cDNA synthesis reaction was performed by using the GoScript Reverse Transcription System (Promega, Madison, WI, USA). The details of the primers and RT-PCR conditions are given in S1 Table. The PCR products were examined by use of 1% agarose gel electrophoresis according to standard procedures. The target fragment was cloned into the pMD18-T vector (Takara, Dalian, China) and sequenced.

The copy number of ZIKV in the positive tissues of mosquitoes was further detected by RT-qPCR using SYBR R select master mix, with amplification by the 7500 Real-Time PCR systems according the manufacturer’s protocol. The details of the primers and RT-qPCR conditions are given in S1 Table. The fragment with 141 bp was cloned into pMD18-T and linearized by EcoRI as previously described [25]. The concentration of plasmid was detected by NanoDrop 2000 Spectrophotometer. A standard curve was established by 10-fold dilutions of the plasmid standard (1.16× 10^2^–1.16× 10^7^). Each sample was conducted in three replicates, and the results were determined by the melting curve and cycle threshold values.

For those mosquitoes with ZIKV-negative midguts, we did not further analyze the ovaries. The ovaries of positive mosquitoes were dissected over the course of two gonotrophic cycles. At 14 dpi, the remaining mosquitoes were transferred into cages with moist filter paper for oviposition. After 3 days, the mosquitoes were fed with pure sheep blood after starvation for 24 h. The midguts and ovaries were dissected, and ZIKV was detected at each of the time points during second gonotrophic cycle (SGC), corresponding to 23, 26, 29, and 33 dpi. The experiment was carried out in triplicate, with 240 samples in total.

### Immunohistochemistry assay

After removal of the legs and wings, forty ZIKV-exposed mosquitoes were harvested at 14 dpi and immediately fixed for 16 h in 10% neutral buffered formalin. The entire body was embedded in paraffin and serially cut for histologic examination. The slides were stained with mouse monoclonal (B4) to ZIKV NS1 protein antibody (Abcam, MA) and horseradish peroxidase (HRP)-conjugated anti-mouse IgG secondary antibody (ZSGB-BIO, China). Following the procedure outlined elsewhere [26], 3,3’-diaminobenzidine (DAB) and hematoxylin (Baso, China) were applied, and the specimens were examined under a light microscope (Nikon, Japan). Tissues dissected from mosquitoes fed on an uninfected blood meal were used as the control.

### Vertical transmission

Starved female mosquitoes were separated into four groups (group I, II, III, and IV, 42 mosquitoes in each group) as shown in S1 Fig. Individual mosquitoes from group I were transferred to a 250-ml paper cup with filter paper after being fed an infectious blood meal. The mosquitoes laid eggs from 4 to 7 dpi, and the eggs were collected. After counting, the eggs on the single filter paper were placed into a 1.5-ml tube, considered one pool, for detecting ZIKV. Immediately after, these mosquitoes were transferred to a new paper cup individually and starved for 24 h. Then, they were refed with pure sheep blood to stimulate oviposition, which continued for 3 days. These eggs were collected in a new 1.5-ml tubes and cold homogenized as mentioned above. The ovaries of the mosquitoes were dissected for detecting ZIKV. Only the eggs coming from mosquitoes with ZIKV-positive ovaries who laid eggs twice were studied.

Mosquitoes from group II were fed only a sheep blood-C6/36 cell mixture as a control, whereas those in group III were provided with a sheep blood-ZIKV mixture as a treatment. The ovaries of the experimental groups were dissected directly for detecting ZIKV after the first oviposition, which continued for 3 days, on the 7th dpi. The eggs from ZIKV-positive ovaries were reared to adulthood. The male and female progeny in the treatment groups were dissected as follows: males: testis and accessory glands; females: salivary glands. The tissues were cold homogenized separately for detecting ZIKV as described above. The eggs of the control groups were also reared to adulthood and examined to compare the hatching, pupation, and emergence rates.

After being fed an infectious blood meal, mosquitoes from group IV were transferred to a 250-ml paper cup separately for oviposition from 4 to 7 dpi. The ovaries of the mosquitoes were dissected for detecting ZIKV. Only the eggs from mosquitoes with ZIKV-positive ovaries were studied. The eggs were collected and reared to adulthood. After 4 days of emergence, 30 female offspring were allowed to feed on one C57BL/6 mouse (one day old) for 1 h. The salivary glands of female offspring were dissected for detecting ZIKV. Mouse blood (200 μl) was collected into a 1.5-ml tube by tail snipping at 1, 3, and 5 dpi. A total of 100 μl of mouse blood was used to detect the copy number of ZIKV. For those with ZIKV-positive blood, we further analyzed the virus titer in another 100 μl of mouse blood by a plaque assay. Suckling mice were euthanized at 7 dpi. Blood from each mouse was collected and evaluated as described above. The experiment was carried out with 13 mice and 390 female mosquito offspring in total.

### Plaque assay

BHK-21 cells (2×10^5^ cells/well) were plated in 12-well plates and incubated in a cell culture incubator until 90% to 95% confluency was reached. After centrifugation, the supernatant of the infectious blood meal or infected mouse blood was inoculated into the wells individually with serial dilutions. Methyl cellulose was used to overlay the infected cell monolayers. Five days after incubation, the cells were fixed with 4% paraformaldehyde solution and then stained with crystal violet solution for 30 min at room temperature. The wells were washed with tap water and dried. The dilution that produced 30 to 100 plaques was selected, and the number of plaques for each replicate was counted. The plaque forming units per ml (PFU/ml) was calculated as follows: PFU/ml = average number of plaques/ [(dilution factor of well)(volume of inoculum per plate)].

### Statistical analyses

All statistical analyses were performed using SPSS version 20.0 (IBM, Chicago, IL, USA). Rates were compared using the chi-square test. The ZIKV RNA copy levels were log-transformed, and the t-test was used to determine differences in viral titers in different tissues among different studies. The number of eggs in the control groups and ZIKV groups was compared using t-test. P-values > 0.05 were considered nonsignificant.

## Results

### ZIKV infection in the ovaries of *Ae*. *albopictus*

**Fig 1:**
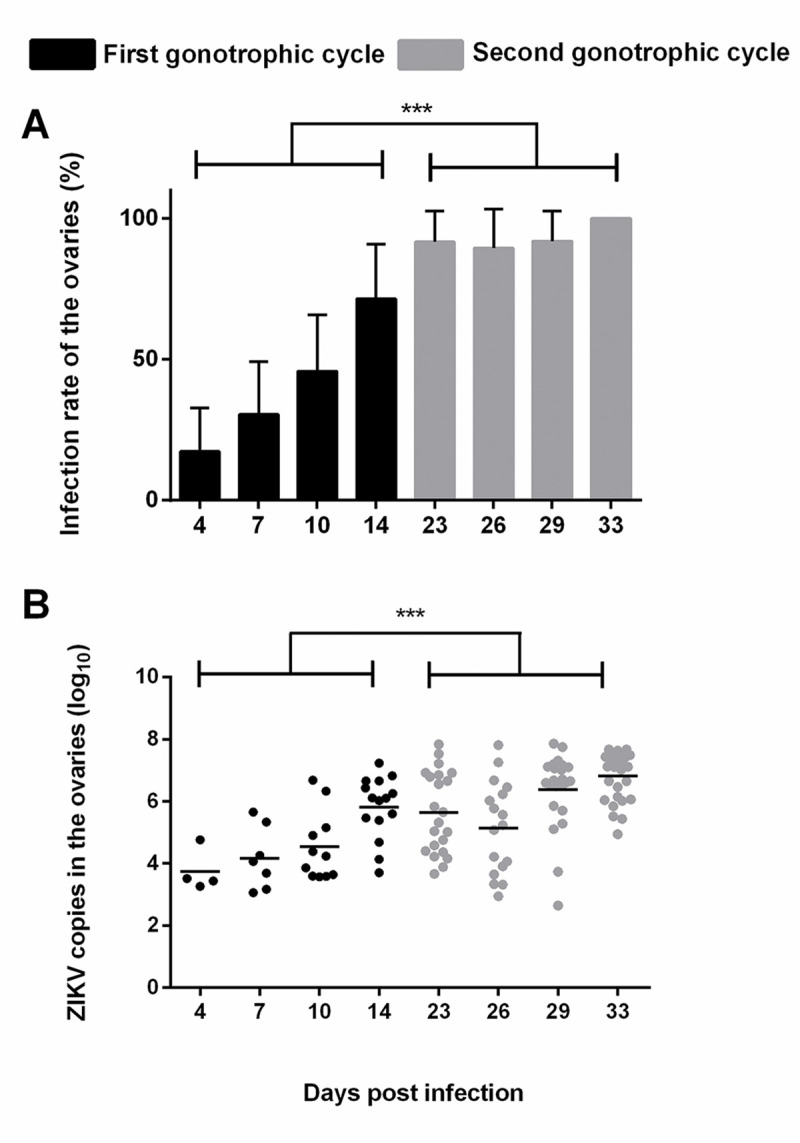
ZIKV infection in the ovaries of *Ae*. *albopictus*. A) Infection rates in the ovaries in the first and secondary gonotrophic cycles. Ovary infection rate: no. of positive ovaries/no. of positive midguts; the error bars represent 95% confidence intervals (CIs); ***, P < 0.001. B) The amount of ZIKV in the ovaries in the FGC and SGC. The titer of ZIKV in the positive ovaries of *Ae*. *albopictus* was detected by RT-qPCR. Horizontal black lines show the median ZIKV copies (log_10_) per ovary. ***, P < 0.001.

A total of 240 *Ae*. *albopictus* females were used to measure the infection rate in the ovaries. RT-PCR and RT-qPCR were used to detect the ZIKV quality and quantity, and IHC was carried out to locate the virus protein in the tissues. At 0 dpi, the infection rate in the midgut of thirty mosquitoes was 100% (S2 Fig), and the RNA (genome) copies of ZIKV in the midguts were 5.02 ± 0.31 (log 10 levels), indicating that all the mosquitoes had ingested the infectious blood meal. Starting from 4 dpi, the ovaries showed evidence of infection and remained positive throughout the remainder of the experiment (Fig 1A). During the FGC, the infection rate of the ovaries was 19.04% at 4 dpi, 30.43% at 7 dpi, 39.28% at 10 dpi and 75% at 14 dpi. During the SGC, the infection rates of the ovaries were similar and reached the maximum value (100%) at 33 dpi. The average infection rate of the ovaries in the SGC (93.75%) was significantly higher than that in the FGC (40.66%) (χ 2 = 60.428, P <0.001).

The amount of ZIKV in the ovaries continually increased during the two gonotrophic cycles (Fig 1B). In the FGC, the log 10 levels remained stable from 3.74 ± 1.35 at 4 dpi to 4.54 ± 2.19 at 10 dpi and then increased to 5.82 ± 1.97 at 14 dpi. In the SGC, the amount of ZIKV was similar at 23 dpi (5.64±2.56) and 26 dpi (5.15±2.92). Thereafter, the titer increased to 6.37±2.40 at 29 dpi and 6.82±1.52 at 33 dpi. The average virus titer in the ovaries in the SGC (6.10±2.60) was higher than that in the FGC (4.90± 2.46) (t = -4.798, p<0.001).

**Fig 2:**
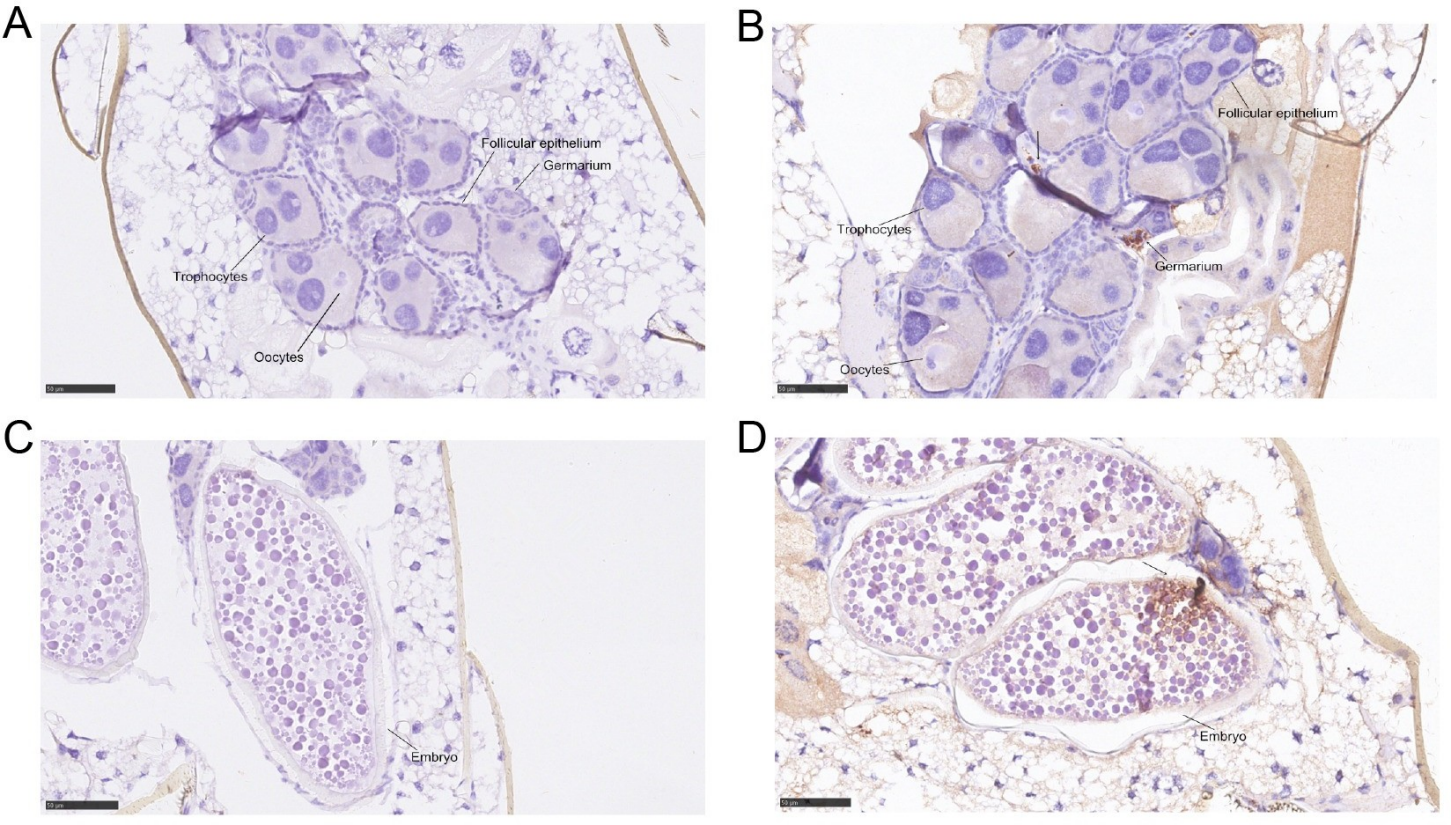
ZIKV infection in the ovaries (14 dpi). Viral antigens were detected using IHC, which revealed viral proteins in the germarium (B) and embryo (D) at 14 dpi. Positive staining for ZIKV is displayed as obvious brownish-red staining caused by the oxidation of DAB by HRP within the organs of the mosquitoes. Tissues that were dissected from uninfected mosquitoes were used as controls (A, C).

In addition to quantitative detection by RT-qPCR, ZIKV antigen was detected by IHC, as indicated by arrows in the germarium of the ovary (Fig 2B) and the embryo (Fig 2D). ZIKV-positive results demonstrated transovarial transmission, a vertical transmission route that is more efficient than transovum transmission. Tissues dissected from uninfected mosquitoes were used as controls (Fig 2A and 2C). No pathogenetic injury was found in the germarium, oocytes, trophocytes and follicular epithelium in the ZIKV-positive ovaries compared with the control ovaries.

### Impact of ZIKV infection on ovarian development and oviposition

The influence of ZIKV on the ovarian development and oviposition of *Ae*. *albopictus* remains unclear. When the ovaries were dissected at 14 and 33 dpi, the end points of the two gonotrophic cycles, and the number of eggs in the ovaries was counted under a microscope to evaluate ovarian development. No mature embryo in the ovary was regarded as undeveloped. The ovaries were collected for detecting ZIKV. For those mosquitoes with ZIKV-negative ovaries, we did not further analyze the ovarian development. To evaluate oviposition, another batch of ZIKV-infected mosquitoes was allowed to lay eggs at 14 and 33 dpi for three days. Ovaries were dissected to count the remaining eggs and detect ZIKV. For those mosquitoes with ZIKV-negative ovaries, we did not further analyze the oviposition. The control group of mosquitoes was fed with an uninfected blood meal as described above.

**Fig 3:**
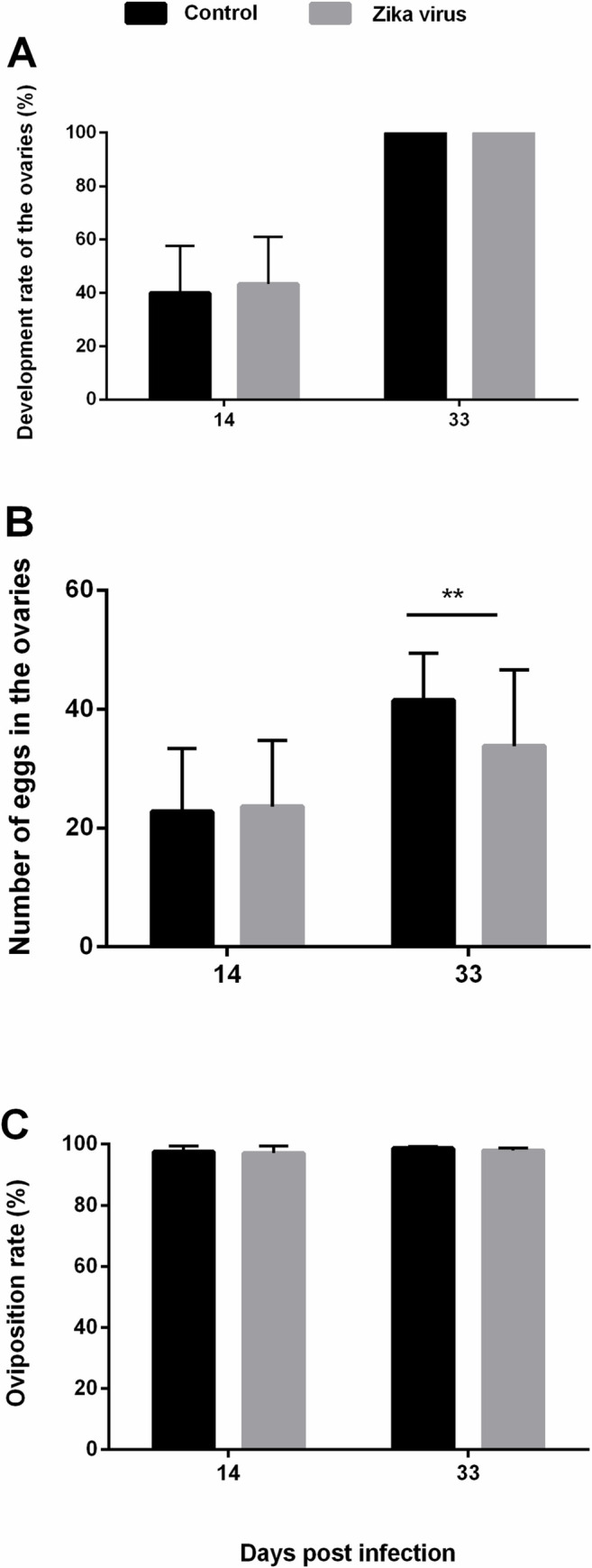
Impact of ZIKV infection on ovarian development and oviposition. Ovarian development and oviposition in *Ae*. *albopictus* were compared with those in the control group at 14 and 33 dpi. (A) Development rate of the ovary: no. of developed ovaries/no. of tested ovaries; the error bars represent 95% CIs. No mature embryo in the ovary was regarded as undeveloped. (B) The number of eggs in the ovaries. Error bars represent SDs. **, *P* < 0.01. (C) Oviposition rate: no. of oviposited eggs/total no. of eggs; the error bars represent 95% CIs.

In this study, the development rates of infected ovaries at 14 and 33 dpi did not show any significant differences from that in uninfected mosquitoes (Fig 3A), which suggested that ZIKV did not affect the ovarian development of mosquito hosts. At 14 dpi, the number of eggs in the ZIKV group was similar to that in the control group (p>0.05). However, at 33 dpi of the SGC, the number of eggs in the control groups was significantly higher than that in the ZIKV groups (Fig 3B) (t = 2.767, P = 0.008). As Fig 3C shows, oviposition rates were calculated, and no significant difference was detected, which suggested that ZIKV may negatively influence fecundity but not oviposition of the infected mosquitoes.

### ZIKV infection and transmission in offspring

**Fig 4:**
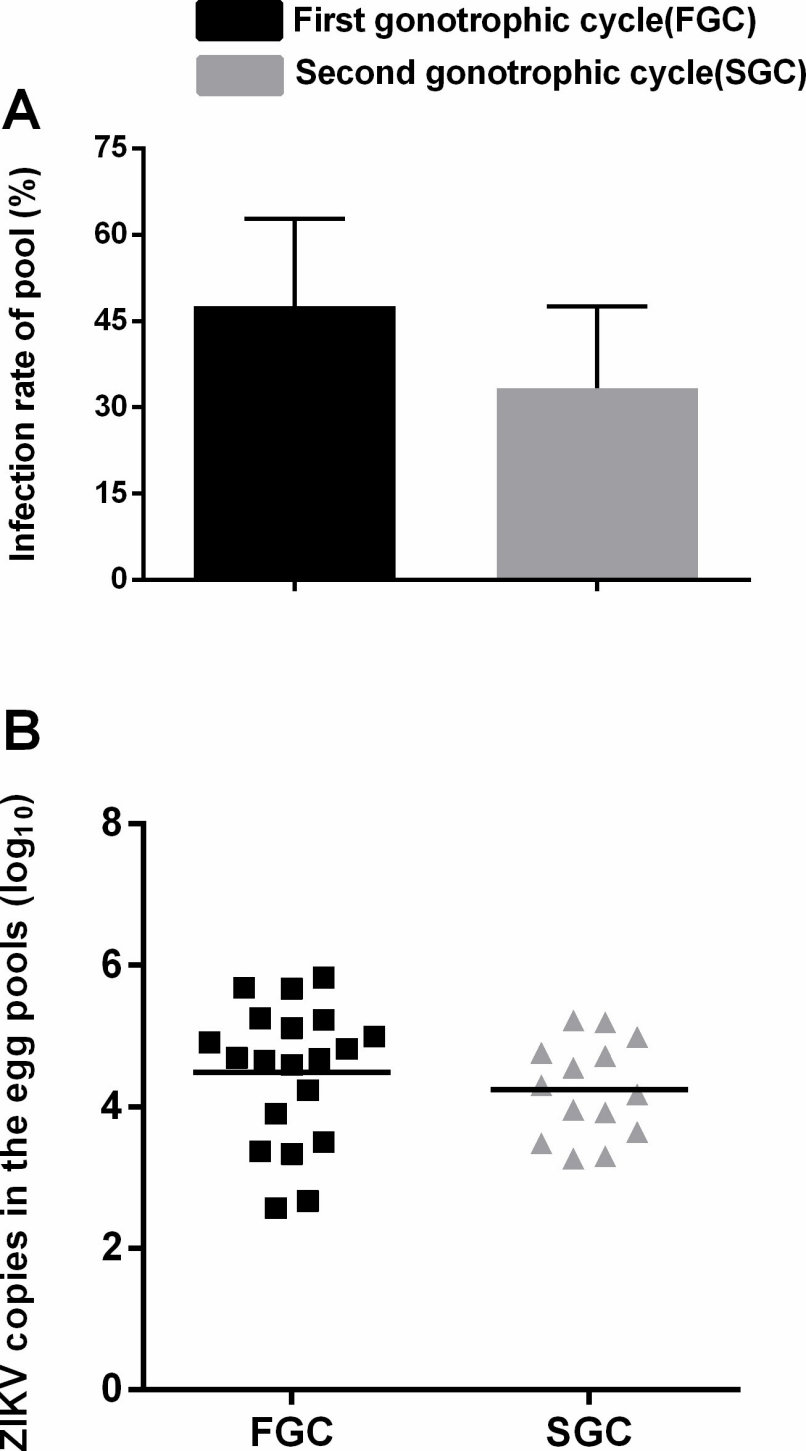
ZIKV infection in F_1_ eggs. A) Infection rates of egg pools in the FGC and SGC. Error bars represent 95% CIs. B) The amount of ZIKV in the egg pools in FGC and SGC. Horizontal black lines show the median ZIKV copies (log_10_) in each tissue.

*Ae*. *albopictus* of the Foshan strain can transmit ZIKV from G_0_ to the adult F_1_ generation, which is clear evidence that this vector mosquito can vertically transmit ZIKV. In this study, the infection rate in the ovaries was 100%. Regarding the egg pools, as shown in Fig 4A, the infection rate in the FGC (47.62%) was not different from that in the SGC (33.33%) (p>0.05). The amount of ZIKV in the egg pools was calculated, and the log 10 levels in the FGC (4.48±1.89) were not different from those in the SGC (4.25±1.33) (Fig 4B) (p>0.05).

To evaluate the influence of ZIKV on offspring development, the rates of hatching, pupation and emergence were compared with those in the control group. In the control group, the rates of hatching, pupation and emergence were 61.01%, 93.08% and 95.15%, respectively, while the rates in the ZIKV group were 57.49%, 91.19% and 98.12%, respectively. No significant difference was found between them. These results suggested that ZIKV infection did not prevent the growth and development of the offspring.

**Fig 5:**
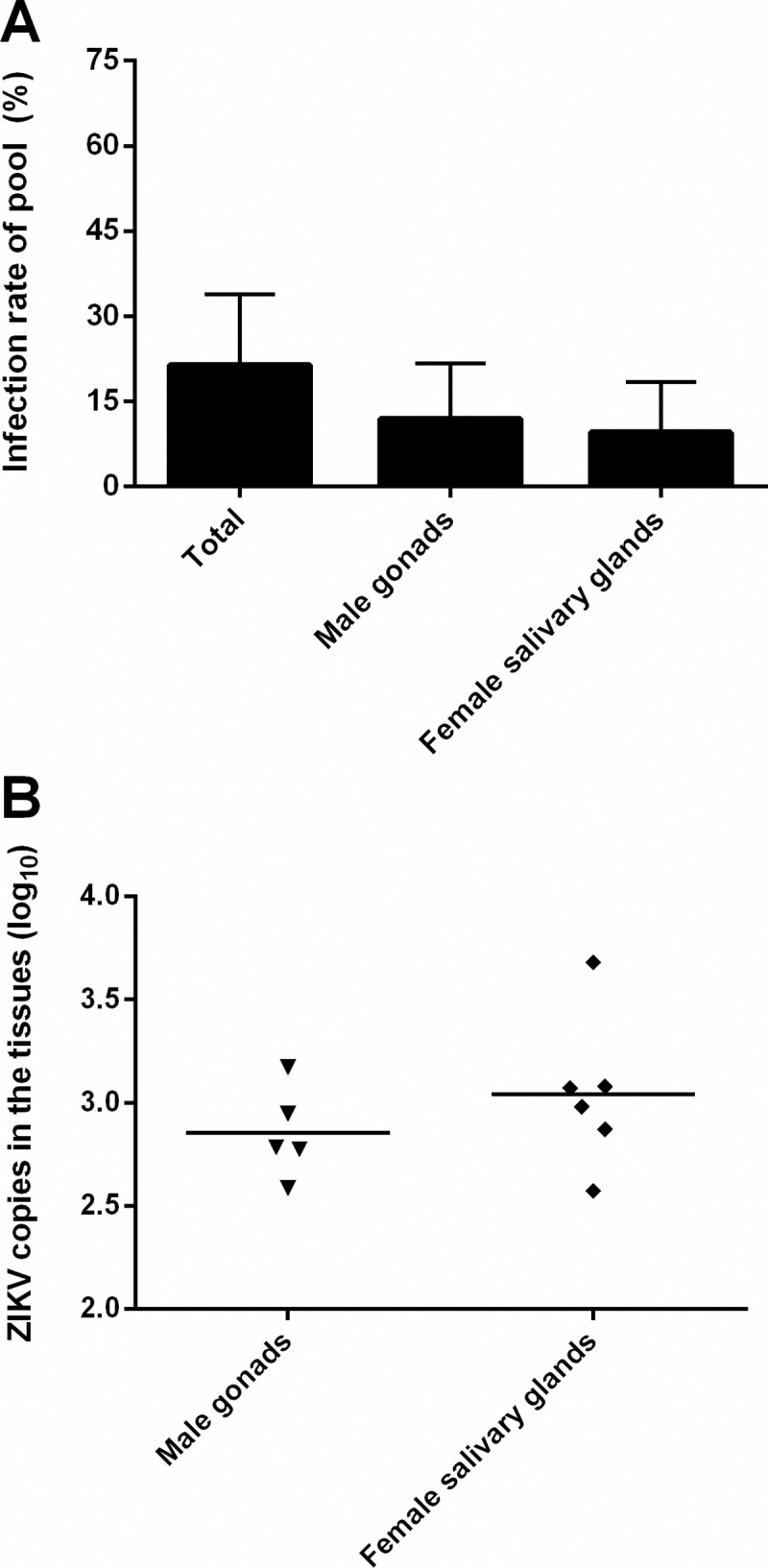
ZIKV infection in F_1_ adults. A) Infection rates of ZIKV in F_1_ adults. Error bars represent 95% CIs. B) The amount of ZIKV in F_1_ adults (male and female). Horizontal black lines show median ZIKV copies (log_10_) in each tissue.

In the adult F_1_ pool, the infection rates of males and females were 11.90% and 9.52%, respectively, and no obvious difference was found (Fig 5A) (p>0.05). The titer of ZIKV was 3.04 ± 0.71 in male gonads and 2.85±0.43 in female salivary glands (Fig 5B) (p>0.05), suggesting that no sex tendency occurred when ZIKV was vertically transmitted to the adult F_1_ generation. However, positive results in organs suggested that ZIKV may initiate new infections by horizontal and venereal transmission within this vector insect population.

To evaluate the efficiency of vertical transmission of ZIKV in *Ae*. *albopictus*, filial infection rates were calculated. As Table 1 shows, the minimal filial infection rates in eggs from two gonotrophic cycles were 2.06% and 0.69%, without statistical significance (p>0.05). In our study, the effective population transmission rate (minimum of 1.87%) was calculated in F_1_ adult tissues instead of F_1_ adult whole bodies; this result indicated that ZIKV can not only vertically transmit to F_1_ eggs but also persistently exist in F_1_ adults, even though the ratio was not high.

**Table 1:** Filial infection rates in eggs and adults.

Exp.	Eggs		Adults		
	FGC	SGC	Male	Female	Total
No. tested	921	2036	296	293	589
No. positive	19*	14*	5	6	11
MIR (%)	2.06†	0.69 ‡	1.69	2.05	1.87§

*No. of ZIKV-positive egg pools.

†Minimal filial infection rate in the FGC = no. of positive egg pools in the FGC/no. of tested eggs.

‡Minimal filial infection rate in the SGC = no. of positive egg pools in the SGC/no. of tested eggs.

§Effective population transmission rate = no. of positive adults/no. of tested adults.

### C57BL/6 Mice Bitten by Vertical Transmitted F_1_ female

To evaluate the vector capacity of the offspring infected by vertical transmission, thirteen C57BL/6 suckling mice were fed on by ZIKV-positive F_1_ females (Table 2). At 1 dpi, ZIKV was undetectable in all the mice. At 3 dpi, ZIKV was detected in three of the thirteen mice. The ZIKV load was 2.13 log10 copies/μl (4.25*10^3^ PFU/ml) in the No. 3 mouse, 2.42 log10 copies/μl in the No. 7 mouse and 2.30 log10 copies/μl (5*10^3^ PFU/ml) in the No. 12 mouse. The ZIKV load of the No. 3 mouse continuously increased and reached 2.56 log10 copies/μl (7.5*10^3^ PFU/ml) at 5 dpi and 3.08 log10 copies/μl (7.5×10^4^ PFU/ml) at 7 dpi. The ZIKV load in the No. 7 mouse was only detected at 3 dpi by RT-PCR and undetected by plaque assay. The ZIKV load in the No. 12 mouse was detected at 3 dpi, 5 dpi and 7 dpi by RT-PCR but could only be detected by plaque assay at 3 dpi (5*10^3^ PFU/ml) and 5 dpi (5.5*10^3^ PFU/ml). These positive results demonstrate that female progeny infected with ZIKV via vertical transmission could transmit the virus to a mammalian host through feeding.

**Table 2:** ZIKV transmission by vertically infected offspring.

Mouse no.	Mosquito			Virus load in mouse blood† (Virus titer ‡)			
	Tested	No. positive	Virus load*	1d	3d	5d	7d
1	30	0	-	-	-	-	-
2	30	1	2.97	-	-	-	
3	30	1	3.56	-	2.13 (4.25*10^3^)	2.56 (7.5*10^3^)	3.08 (7.5*10^4^)
4	30	1	3.42	-	-	-	-
5	30	0	-	-	-	-	-
6	30	0	-	-	-	-	-
7	30	2	3.41&2.71	-	2.42 (N)	-	-
8	30	0	-	-		-	-
9	30	1	2.27	-	-	-	-
10	30	1	3.37	-	-	-	-
11	30	0	-	-			-
12	30	1	2.62	-	2.30 (5*10^3^)	2.68 (5.5*10^3^)	2.41 (N)
13	30	0	-	-	-	-	-

* Copy number of ZIKV in F_1_ female offspring (log10 copies/μl)

† Copy number of ZIKV in the mouse blood (log10 copies/μl)

‡ Vitus titer in the mouse blood (PFU/ml)

N = Negative

## Discussion

In this study, we experimentally demonstrated that *Ae*. *albopictus* could transmit ZIKV vertically from a female parent to her F_1_ progeny (eggs and adults). Vertically infected F_1_ female offspring transmitted ZIKV to C57BL/6 suckling mice via mosquito bites, which is the first evidence that female offspring infected through vertical transmission can initiate a new horizontal cycle.

Considering the results of other studies on vertical transmission of flavivirus pathogens in mosquitoes [5–8, 27], the detection of ZIKV vertical transmission to offspring by infected *Ae*. *albopictus* females is not unexpected. In our study, the minimal filial infection rates of eggs from two gonotrophic cycles (2.06% and 0.69%) were close, which is similar (1.18%) to results involving *Ae*. *albopictus* in Alexander et al.’s report [14]. However, in a previous report, minimal filial infection rates of ZIKV in *Ae*. *aegypti* were 5.49% in larvae, 5.00% in pupae and 6.99% in adults [15], which could be explained by the fact that *Ae*. *aegypti* is a better vector than *Ae*. *albopictus* [20, 25]. For dengue virus (DENV), another mosquito-borne flavivirus that poses a serious threat worldwide, filial infection rates in three strains of *Ae*. *albopictus* from Brazil ranged from 0.16% to 0.52% in males and from 0.32% to 0.53% in females [28]. However, in another report, filial infection rates of DENV in five geographical strains of *Ae*. *albopictus* ranged from 0.52% to 2.89%, and filial infection rates of individual positive families within the strains ranged from 1.4% to 17.4% [29]. These conflicting results might be attributable to different genetic backgrounds, geographic regions, arbovirus strains or vector population dynamics.

In adult mosquitoes, the effective population transmission rate (minimum of 1.87%) was calculated in this study. No difference was found between male (11.90%) and female offspring (9.52%) in infection rate or the amount of ZIKV (Fig 5); however, a previous report showed that female offspring (13.17%) had a higher infection rate than male offspring (7.88%) [30]. Research on the sex tendency of infection between male and female offspring is scarce. Importantly, congenitally infected females are able to carry the virus for their entire life and transmit the virus horizontally by feeding without the requirement for an extrinsic incubation period (approximately 8 to 14 days) [31]. Considering this situation, the low filial infection rates indicated that vertical transmission is an alternate transmission mechanism, and ZIKV might use this mechanism to maintain in a vector population, which could play a significant role in establishing endemicity.

Vertical transmission may occur through two main mechanisms: transovarial transmission and transovum transmission. The IHC results in this study provide the first evidence of the virus location, indicating that the route of vertical transmission of ZIKV in *Ae*. *albopictus* is transovarial transmission, which is more efficient than transovum transmission [27]. When ZIKV enters the ovary, the germariums become infected. In each subsequent gonotrophic cycle, a new follicle breaks away from the germarium and develops to maturity [32]. The virus replicates within the embryo and persistently exists in the eggs and adult offspring. Since the minimum filial infection rates detected from surface-sterilized eggs were similar to those obtained from unsterilized eggs, Barry et al. concluded that the vertical passage of yellow fever virus (flavivirus) in *Ae*. *aegypti* was transovarial rather than transovum [33]. In another study involving DENV, IFA-positive germarium and follicles demonstrated the potential for transovarial transmission in *Ae*. *aegypti* [34]. However, transovum transmission of DENV, Japanese encephalitis viruses and St. Louis encephalitis virus by *Ae*. *albopictus* occurs when the fully formed egg enclosed in the chorion is oviposited [35]. These two mechanisms of vertical flavivirus transmission may exist simultaneously in *Aedes* mosquitoes.

As the result of IHC showed, ZIKV did not cause pathological damage in the ovary. The number of eggs in the ovaries decreased after ZIKV infection at 33 dpi in the SGC, implying that a high level of ZIKV infection did not inhibit ovarian development and oviposition but negatively influenced oogenesis. In a previous study, a negative relationship between the body titer of *Ae*. *albopictus* with nondisseminated DENV infection and the number of eggs laid was recognized [36]. These results implied that flaviviruses (ZIKV and DENV) may negatively influence oogenesis in *Ae*. *albopictus*. However, fecundity and fertility (the number of viable offspring produced) were evaluated in ZIKV-positive *Ae*. *aegypti*, and no significant difference was found [37]. These conflicting results may be due to differences in the experiments and require further study for clarification. When comparing the rate of hatching, pupation, and emergence with those in the control groups, ZIKV did not prevent completion of the mosquito’s life cycle.

In this study, three of thirteen C57BL/6 suckling mice were positive, and ZIKV positivity persisted for up to seven days in the blood after one suckling mice was bitten by F_1_ female offspring. Prior studies confirmed that ZIKV and DENV can be transmitted vertically to female offspring; antigens were detected in F_1_ salivary glands [17] and saliva [38] but not in mammal hosts. C57BL/6 mice are immunocompetent mammals that are commonly used in the laboratory. The virus titer in the first positive mouse continuously increased and reached 7.5×10^4^ PFU/ml at 7 dpi. ZIKV was only detected at 3 dpi in the second positive mouse by plaque assay. In the third positive mouse, the viremia persisted at least for 5 days. The present result suggests that vertically transmitted ZIKV may play a role in disease transmission.

In conclusion, ZIKV can be vertically transmitted in *Ae*. *albopictus* via transovarial transmission. The vertical transmission rates in F_1_ eggs and adults were 2.06% and 1.87%, respectively. Even though the vertical transmission rates were low, the female mosquitoes infected via the congenital route horizontally transmitted ZIKV to immunocompetent mice through feeding. The present study deepens the understanding of the vertical transmission of flaviviruses in *Aedes* mosquitoes and sheds light on the prevention and control of mosquito-borne diseases.

## Supporting information

S1 Fig Schematic representation of the experimental design (vertical transmission). (TIF)

S2 Fig ZIKV infection in the midguts of *Ae*. *albopictus*. Infection rates in the midguts in the first and secondary gonotrophic cycles. Midgut infection rate: no. of positive midguts/total no. of midguts; the error bars represent 95% CIs; NS, P > 0.05.
(TIF)

S1 Table Primer used for amplification and sequencing of Zika virus. (DOC)
