# Supplementary figures and images for: Vertical transmission of zika virus in Aedes albopictus
Source: PLoS Negl Trop Dis. 2020 Oct 15;14(10):e0008776. doi: 10.1371/journal.pntd.0008776 (PMC7671534; doi:10.1371/journal.pntd.0008776)

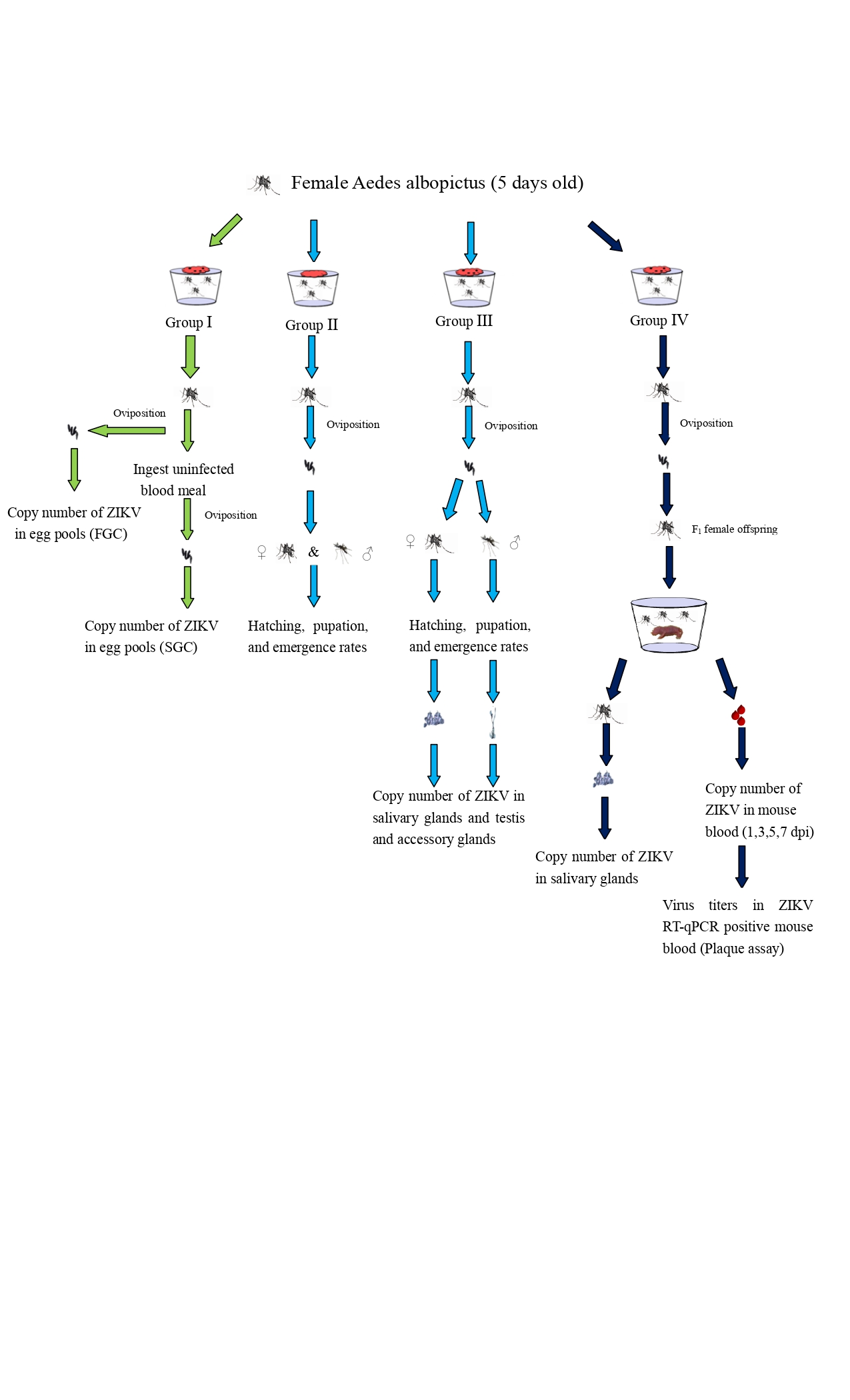

Supplement: S1 Fig — (TIF) [file pntd.0008776.s001.tif]

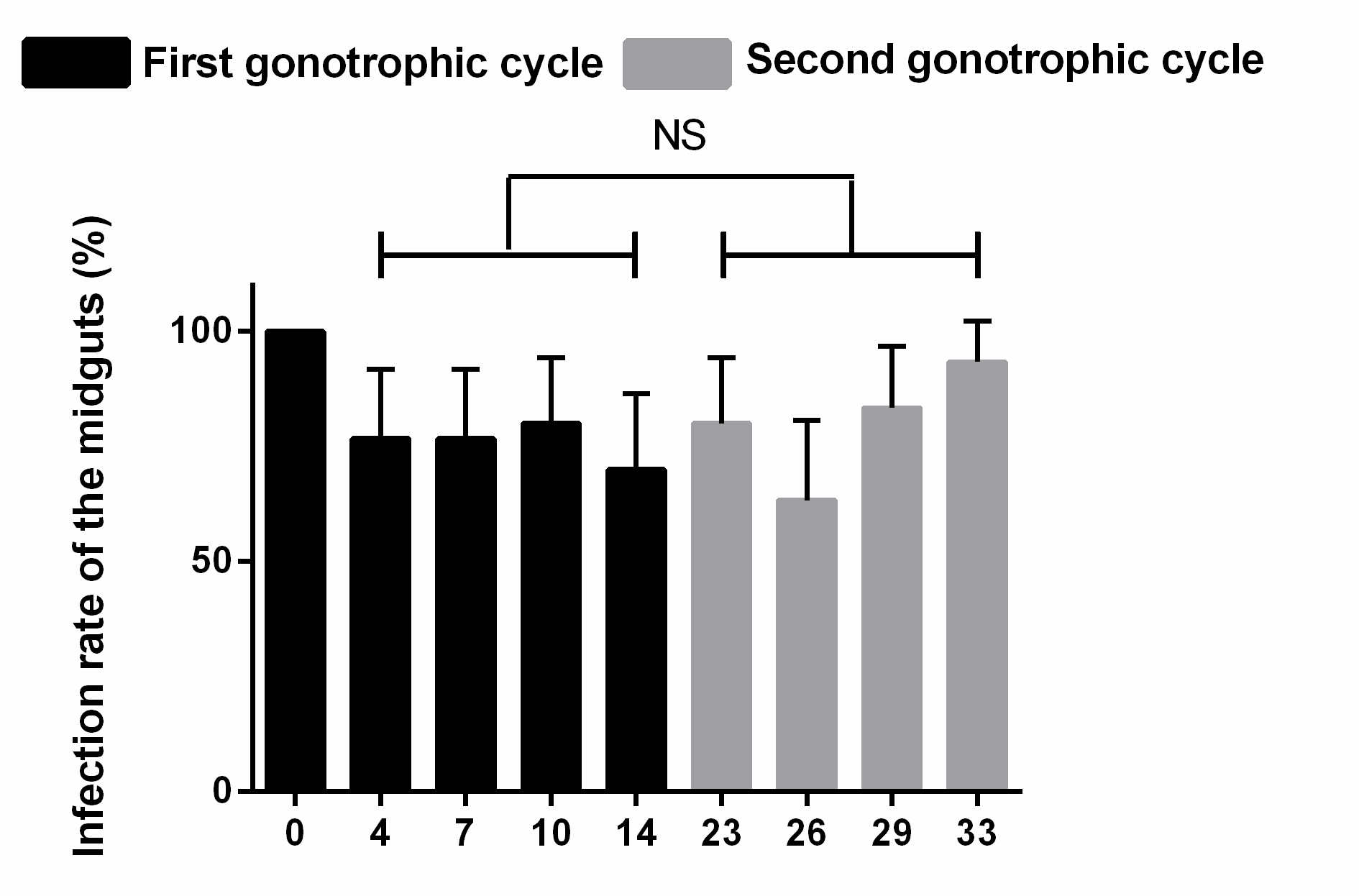

Supplement: S2 Fig — Infection rates in the midguts in the first and secondary gonotrophic cycles. Midgut infection rate: no. of positive midguts/total no. of midguts; the error bars represent 95% CIs; NS, P > 0.05. (TIF) [file pntd.0008776.s002.tif]
