# Supplementary material for: Vertical transmission of zika virus in Aedes albopictus
Source: PLoS Negl Trop Dis. 2020 Oct 15;14(10):e0008776. doi: 10.1371/journal.pntd.0008776 (PMC7671534; doi:10.1371/journal.pntd.0008776)
Supplement: S1 Table — (DOC) [file pntd.0008776.s003.doc]

**S1Table. Primer used for amplification and sequencing of Zika virus. (DOCX)**

| length | Location | Primers (5’-3’) | Condition |
| --- | --- | --- | --- |
| 296bp | Nonstructural protein 1 (NS1) | ACCCAAGTCTTTAGCTGGGC | RT-PCR Initial denaturation at 94°C for 3 min, followed by 35 cycles of 94°C for 30 s, 60°C for 30 s, 72°C  for 30 s, and 72°C for 7 min |
|  |  | CTGGTTCTTTCCTGGGCCTT |  |
| 141bp | Across capsid and propeptide regions | GGAGAAGAAGAGACGAGGCG | RT-qPCR preheat at 50°C for 2 min, 95°C for 2 min; 40 cycles at 95°C for 15 s, 60°C for 15 s, and 72°C for 1 min |
|  |  | GATATGGCCTCCCCAGCATC |  |
